# Understanding COVID-19 Vaccine Confidence in People Living with HIV: A pan-Canadian Survey

**DOI:** 10.1007/s10461-023-03991-8

**Published:** 2023-02-04

**Authors:** Cecilia T. Costiniuk, Joel Singer, Judy Needham, Yanbo Yang, Hong Qian, Catharine Chambers, Ann N. Burchell, Hasina Samji, Ines Colmegna, Sugandhi del Canto, Guy-Henri Godin, Muluba Habanyama, Christian Hui, Abigail Kroch, Enrico Mandarino, Shari Margolese, Carrie Martin, Maureen Owino, Tima Mohammadi, Wei Zhang, Sandra Pelaez, Colin Kovacs, Erika Benko, Branka Vulesevic, Curtis L. Cooper, Aslam H. Anis

**Affiliations:** 1 Deparment of Medicine, Division of Infectious Diseases and Chronic Viral Illness Service, McGill University Health Centre, Montreal, QC Canada; 2 Infectious Diseases and Immunity in Global Health Program, Research Institute of McGill University Health Centre, Montreal, QC Canada; 3 School of Population and Public Health, University of British Columbia, Vancouver, BC Canada; 4 Centre for Health Evaluation and Outcome Sciences, St. Paul’s Hospital, Vancouver, BC Canada; 5 CIHR Canadian HIV Trials Network, Vancouver, BC Canada; 6 Faculty of Medicine, McGill University, Montreal, QC Canada; 7 Dalla Lana School of Public Health, University of Toronto, Toronto, ON Canada; 8 MAP Centre for Urban Health Solutions, Li Ka Shing Knowledge Institute, St Michael’s Hospital, Unity Health, Toronto, ON Canada; 9 Department of Family and Community Medicine, Faculty of Medicine, University of Toronto, Toronto, ON Canada; 10 British Columbia Centre for Disease Control, Vancouver, BC Canada; 11 Faculty of Health Sciences, Simon Fraser University, Burnaby, BC Canada; 12 Division of Rheumatology, Department of Medicine, McGill University Health Centre, Montreal, QC Canada; 13 Community Advisory Committee, CIHR Canadian HIV Trials Network, Vancouver, BC Canada; 14 Policy Studies, Toronto Metropolitan University, Toronto, ON Canada; 15 Ontario HIV Treatment Network, Toronto, ON Canada; 16 School of Kinesiology and Physical Activity Sciences, Faculty of Medicine, University of Montreal, Montreal, QC Canada; 17 Division of Infectious Diseases, Faculty of Medicine, University of Toronto, Toronto, ON Canada; 18 The Ottawa Hospital, Ottawa Hospital Research Institute, Ottawa, ON Canada; 19 Research Institute of the McGill University Health Centre, 1001 boul Decarie, Block E, Room EM2, 3226, H4A 3J1 Montreal, QC Canada; 20 The Maple Leaf Medical Clinic, Toronto, ON Canada

**Keywords:** HIV, intention to vaccinate, COVID-19 vaccine, vaccine confidence, vaccine hesitancy, survey

## Abstract

Understanding the roots of Covid-19 vaccine hesitancy in at-risk groups, such as persons living with HIV (PLWH), is of utmost importance. We developed a modified Vaccine Hesitancy Scale (VHS) questionnaire using items from the National Advisory Committee on Immunization Acceptability Matrix. To examine factors associated with receiving COVID-19 vaccine and the link between vaccine attitudes and beliefs with vaccine behavior, PLWH were recruited via social media and community-based organizations (February-May 2022). Descriptive statistics were used to summarize results. Total VHS score was generated by adding Likert scale scores and linear regression models used to compare results between participants who received or did not receive COVID-19 vaccines. Logistic regression models were used to identify factors associated with vaccine uptake. A total of 246 PLWH indicated whether they received a COVID-19 vaccine. 89% received ≥ 1 dose. Mean total VHS(SD) for persons having received ≥ 1 COVID-19 vaccine was 17.8(6.2) vs. 35.4(9.4) for participants not having received any COVID-19 vaccine. Persons who received ≥ 1 dose were significantly older than those who had not received any (48.4 ± 13.8 vs. 34.0 ± 7.7 years, p < 0.0001). The majority of participants considered COVID-19 vaccination important for their health(81.3%) and the health of others(84.4%). Multivariate logistic regression revealed the odds of taking ≥ 1dose were increased 2.4-fold [95% CI 1.6, 3.5] with each increase in age of 10 years (p < 0.0001). Sex and ethnicity were not different between groups. In conclusion, PLWH accept COVID-19 vaccines for both altruistic and individual reasons. With evolving recommendations and increasing numbers of booster vaccines, we must re-examine the needs of PLWH regularly.

**Supplementary Information:**

The online version contains supplementary material available at 10.1007/s10461-023-03991-8.

## Introduction

Approximately 37 million people are living with HIV (PLWH) worldwide UNAIDS. 38 million people are living with HIV around the world. https://www.unaids.org/en/resources/infographics/people-living-with-hiv-around the world. Acccessed 6 June 2022[1], of whom ~ 67,000 reside in Canada Public Health Agency of Canada Estimates of HIV incidence, prevalence and Canada’s progress on meeting the 90-90-90 targets https://www.canada.ca/en/public-health/services/publications/diseases-conditions/summary-estimates-hiv-incidence-prevalence-canadas-progress-90-90-90.html#s2, Accesssed 6 June 2022[2]. Effective antiretroviral therapy (ART) dramatically reduced morbidity and mortality associated with HIV infection Palella FJ, Jr., Delaney KM, Moorman AC, Loveless MO, Fuhrer J, Satten GA, et al. Declining morbidity and mortality among patients with advanced human immunodeficiency virus infection. HIV Outpatient Study Investigators. N Engl J Med. 1998;338(13):853-60. Hogg RS, Heath KV, Yip B, Craib KJ, O'Shaughnessy MV, Schechter MT, et al. Improved survival among HIV-infected individuals following initiation of antiretroviral therapy. JAMA. 1998;279(6):450-4. 2022[3,4]. and lowered the rate of opportunistic infections and associated deaths. People with advanced HIV, those with low CD4 T-cell counts and high HIV viral loads and those who are not on ART have increased risk of COVID-19-associated complications. Higher rates of chronic lung disease and behaviors that impact lung function (cigarette smoking, drug and alcohol use) among PLWH [1–3] are proposed to increase their COVID-19 risk. Also, the fact that HIV disproportionately affects persons of racial and ethnic minorities and lower sociodemographic status[4] can contribute to disparities in COVID-19 incidence and outcomes[5, 6].

Effective and safe vaccines against SARS-CoV-2 (COVID-19 vaccines) were developed and distributed with an unprecedented speed. Canada launched its COVID-19 vaccine roll-out in December 2020 for adults, with eligibility expanding to include all individuals 12 + years of age (without contraindications) by June 2021[7]. However, since the roll-out, PLWH were not prioritized for vaccination unless individuals met other priority population criteria.

The success of any vaccination program depends on high and persistent vaccine uptake, guided by confidence in the vaccine program and its effectiveness. Even before the COVID-19 global vaccination effort, vaccine hesitancy emerged as a public health challenge[8]. The prevalence of COVID-19 vaccine hesitancy globally was estimated at 8–15%[9–13] and approximately 20% in Canada in 2021[14]. The reasons are dynamic and have changed over time[14, 15]. Vaccine hesitancy is a multi-dimensional construct with various underlying factors[16].

### Determinants of Vaccine Hesitancy and Confidence

Vaccine confidence describes beliefs about vaccination (knowledge), which relate to the attitude towards vaccination[17], while hesitancy refers to delay in acceptance or refusal of vaccination despite the availability of vaccination services[18]. Various models exist to describe the determinants of vaccine hesitancy and/or confidence. Examples include the Theory of Planned Behavior, the Health Belief Model, the “3 C model,” and the “Working Group Determination of Vaccine Hesitancy Matrix”[14, 15, 18, 19]. The Theory of Planned Behavior aims to provide a rationale for behaviors over which people can exert self-control[20, 21]. The key component to this model is behavioral intent, in that behavioral intentions are influenced by the attitude about the likelihood that the behavior will have the expected outcome and the weighing of the risks and benefits of that outcome. This model also stipulates that behavioral achievement depends on both motivation (intention) and ability (behavioral control) [20, 21]. Meanwhile, according to the Health Belief Model, the two components of health-related behavior are 1) the desire to avoid illness, or conversely get well if already ill; and 2) the belief that a specific health action will prevent, or cure, illness[22, 23]. Ultimately, an individual’s course of action often depends on the person’s perceptions of the benefits and barriers related to health behavior[22, 23]. In the “3 C model,” determinants include 1) *confidence*, which refers to the issues of trust in the safety and effectiveness of vaccines and the competence of health care providers, health care systems and policy makers; 2) *convenience*, which refers to the ease at which vaccines are accessed; and 3) *complacency*, which occurs when the need to vaccinate is low due to perceived low risk of vaccine preventable disease[18, 24]. In later years, additional determinants have been added to this model including *calculation*, which occurs when individuals seek out information and assess their findings to guide decision making, and *collective*, which describes when individuals make decisions based on their sense of social responsibility[18, 24]. Meanwhile, in the “Working Group Determination of Vaccine Hesitancy Matrix,” determinants include 1) contextual influences, 2) individual and group influences and 3) vaccine and vaccination-specific issues[25]. Each category contains specific items which gives details about their determinants and the specific scope[25]. These large theoretical constructs can be used to ground Vaccine Hesitancy Models. In a review of 15 studies by Troiano et al., COVID-19 vaccine acceptance or refusal was associated with ethnicity, working status, religious beliefs, politics, gender, age, education and income[16]. The most common reasons for COVID-19 vaccine refusal included: being against vaccines in general, concerns about safety/thinking that the vaccine was produced in a rush and was too dangerous, or considering the vaccine useless because of the harmless nature of COVID-19, general lack of trust, doubts about the efficiency of the vaccine, belief to be already immunized, and doubts about the vaccine provenience[26].

### Underlying Reasons for Vaccine Confidence and Hesitancy in PLWH

Attitudes towards vaccination result from a complex interaction between different social, cultural and personal factors. Factors facilitating willingness to be vaccinated amongst high-risk populations included personal susceptibility[27]. For Black, indigenous and people of colour (BIPOC) communities, vaccine hesitancy is partially rooted in systemic racism, marginalization and neglect[11]. Stigma, defined as a societal process leading to devaluation or discrediting based on particular attributes, is recognized as a barrier to protective health behaviors across a range of health conditions, including vaccine hesistancy[28]. Medical mistrust is defined as active distrust in health care systems and medical providers with the belief that they are acting against one’s best interest[29]. Nearly all PLWH (97%) in a study from the USA held at least one COVID-19 vaccine or treatment hesitancy belief[30]. Recognizing that PLWH face additional barriers to health care including stigma, discrimination and isolation, Rodriguez et al. found that “lack of confidence” and “risks” explained 46% and 12% of the variance between individuals accepting or refusing the vaccine[31]. At the current time, reasons for COVID-19 vaccine hesitancy and confidence in PLWH in Canada are only starting to be examined, and interventions to increase COVID-19 vaccine uptake in this population have not yet been implemented. Identification of facilitators underlying vaccine confidence among PLWH may reduce health disparities which could be exacerbated by COVID-19 and will guide intervention strategies, such as educational interventions targeted toward specific sub-populations. Given the unique characteristics of PLWH, whose reasons for vaccine refusal may differ from those in the general population, we aimed to address this gap of knowledge in the Canadian context.

### Study Objective

We aimed to identify factors associated with COVID-19 vaccine uptake versus vaccine refusal in PLWH in Canada and determine factors related to confidence in COVID-19 vaccines in order better tailor vaccine recommendations to PLWH.

## Methods

### Study Design and Participants

We carried out a national online survey of PLWH across Canada. In an attempt to ensure a representative sample[32], we aimed to recruit a minimum number of participants who identified with various group membership: men who have sex with men (MSM), people who inject drugs, women, persons of African, Caribbean or Black communities, persons of indigenous communities and persons ≥ 65 years of age.

### Strategies for Recruitment

Participants were recruited between February to May 2022 via social media and through community-based organizations serving PLWH. Approximately fifteen clinics or community-based organizations serving PLWH agreed to distribute the survey. Some clinics also put up poster advertisements and provided social media cards in their offices/clinics. As we wanted to hear from as many PLWH as possible, we did not use recruitment quotas for different sub-populations of PLWH. However, in an attempt to ensure a diverse sample of PLWH in Canada, we aimed to recruit a minimum number of participants (at least 20 per group) who identified with particular group membership.

The public survey link was disseminated on community organizations websites and was directly sent to their members via e-mail, e-posters or other social media. Participants were invited to share their thoughts on COVID-19 vaccination by completing an online survey to help us understand factors that contribute to COVID-19 vaccine uptake or refusal among PLWH. It was indicated that this anonymous survey would take about 15 min to complete, and that all adults living with HIV in Canada could take the survey. Furthermore participants were offered the chance to enter a draw to win one of twenty $50 gift cards.

### Study Procedures

After providing consent to participate, screened participants completed the questionnaire online, using a secure, web-enabled survey application (REDCap), hosted on the University of British Columbia network. Data was saved on the CIHR Canadian HIV Trials Network servers at the St Paul’s Hospital in Vancouver, British Columbia. All data were encrypted.

### Questionnaire

Our study was founded on The Theory of Planned Behavior, which aims to provide a rationale for behaviors over which people can exert self-control[20, 21]. A key component of this model is behavioral intent, in that behavioral intentions are influenced by the attitude about the likelihood that the behavior will have the expected outcome and the weighing of the risks and benefits of that outcome [20, 21]. The questionnaire evaluated factors associated with COVID-19 vaccine beliefs and acceptance among PLWH. Vaccine hesitancy is generally associated with a lower compliance with immunization[33]. The study questionnaire was based on instruments developed in several previous studies[25, 34], including those involving PLWH [31, 35, 36]. We used items validated in another study of PLWH since an HIV diagnosis could modify a person’s perception of disease susceptibility and severity. For example, some PLWH may perceive themselves to be at greater risk of COVID-19 acquisition due to having an immunocompromised status. This perception, in turn, could influence their attitudes towards vaccination. In addition, since many PLWH have traditionally encountered stigma due to their HIV status[37], past experiences may impact their perception of stigma related to COVID-19 acquisition, which could also influence their attitudes towards vaccination. Similarly, many sub-populations of PLWH, including individuals born outside of Canada, may have high levels of mistrust in messages from government and health care providers with regards to messaging related to vaccination[38]. The items in the questionnaire contain those included in the validated Acceptability Matrix which focuses on factors demonstrated to have the highest impact on vaccine uptake. These included: (a) perception of vaccine safety and efficacy, (b) perception of disease susceptibility and severity, (c) process of vaccination and (d) knowledge, attitudes and trust [39]. Furthermore, the questionnaire contains a modified Vaccine Hesitancy Scale (VHS) for PLWH toward the COVID-19 vaccine which has acceptable reliability, internal consistency and construct validity[31]. Ten questions/items from the VHS were included using a five-point Likert scale ranging from 1 (strongly agree) to 5 (strongly disagree). The full list of questions can be found in Supplemental Table 1. Participants also answered a series of additional demographic questions. After collection, variables were checked for fraudulent participants or data.

### Primary Endpoint

The primary endpoint was vaccine uptake of at least one dose of COVID-19 vaccine (yes/no).

### Sample Size

We aimed to enroll 250 participants in the study with the expectation that about 200 would have accepted the COVID-19 vaccine and 50 participants would have refused (4:1 ratio), which reflects the level of vaccine uptake in the general Canadian population as of 2021 (80%)[40]. In our protocol, we assumed a standard deviation of 4. We determined that we would have at least 80% power to detect a 2-point difference assuming a standard deviation of 4 and a ratio of 4:1 of those accepting, compared to those refusing, vaccine. In an attempt to capture a diverse sample of PLWH in Canada, we recorded how each participant identifies with particular group membership(s). We decided not to set quotas for specific subpopulations in an effort to allow everyone who wanted to participate the opportunity to do so.

### Data Analyses

Descriptive statistics were used to describe the characteristics of the study participants. For items on the 5-point Likert scale, the scores for each participant were added together (reversing for direction, as necessary). The scores of the ten items were added together to generate a total VHS score[31, 41] for participants who provided a valid response on the ten questions/items. A linear regression model was constructed to compare the VHS total score between participants who took the COVID-19 vaccine and those who refused. The model was adjusted for age and sex as confounding variables. Logistic regression models were used to identify factors associated with COVID-19 vaccine uptake and refusals, Univariate analysis was performed on potential factors. Furthermore, multivariable logistic regression model were created. Age, sex and statistical significant factors from univariate analysis were included in the multivariable logistic regression model. Two-sided p-value of less than 0.05 were considered to indicate statistical significance. Analyses were conducted using SAS version 9.4.

### Ethics

This study was conducted according to the Tri-Council Policy Statement Version 2 (TCPS2) and the principles in the Declaration of Helsinki. Ethical approval was received from the University of British Columbia Providence Health Care Research Ethics Board (H21-03432).

## Results

### Baseline Characteristics and COVID‑19 Vaccine Uptake

A total of 259 individuals participated in the online survey and 246 indicated whether they were vaccinated. The data collection flow chart can be found in Supplement methods (Supplemental Fig. 1). Characteristics of participants and COVID-19 vaccine uptake for each level of a categorical variable can be seen in Table 1 and Supplemental Table 2. The mean age (± standard deviation) was 47 ± 14 years and 73% were male. 80% had completed at least some high school. 65% were born in Canada, 53.7% declared themselves as being white and 44.4% as belonging to the BIPOC community. 52% were diagnosed with HIV for more than 15 years. In the overall sample, 84.5% 84.% This should read 84.5% reported receiving at least one dose of COVID-19 vaccine.

**Table 1:** COVID-19 Vaccine uptake by participant characteristics

	All responders n = 259	Vaccinated n = 219, 84.5%	Unvaccinated n = 27, 10.4%	PNTA* n = 3, 1.1%	Missing n = 10, 4%
Age, mean (SD), year	46.8 (14.0)	48.4(13.8)	34.0 (7.7)	37.0 (7.2)	47.1(15.6)
Age ≥ 40 years old	175	162 (92.6)	5 (2.9)	1 (0.6)	7 (4.0)
Sex					
Female	69	57 (82.6)	9 (13.0)	1 (1.4)	2 (2.9)
Male	189	162 (85.7)	18 (9.5)	2 (1.1)	7 (3.7)
Highest education level					
Less than HS**	5	4 (80.0)	1 (20.0)	0 (0.0)	0 (0.0)
Some/completed HS	55	36 (65.5)	14 (25.5)	1 (1.8)	4 (7.3)
Some/completed university	150	137 (91.3)	6 (4.0)	2 (1.3)	5 (3.3)
Some/completed graduate education	47	41 (87.2)	6 (12.8)	0 (0.0)	0 (0.0)
Total household income					
$29,999 under	83	71 (85.5)	7 (8.4)	1 (1.2)	4 (4.8)
$30,000–59,999	77	64 (83.1)	10 (13.0)	1 (1.3)	2 (2.6)
$60,000 to 89,999	36	29 (80.6)	6 (16.7)	1 (2.8)	0 (0.0)
$90,000 and up	47	41 (87.2)	3 (6.4)	0 (0.0)	3 (6.4)
Current employment status					
Employed	139	120 (86.3)	16 (11.5)	1 (0.7)	2 (1.4)
Unemployed	113	95 (84.1)	10 (8.8)	1 (0.9)	7 (6.2)
Born in Canada	165	132 (80.0)	25 (15.2)	2 (1.2)	6 (3.6)
Inject drugs user	17	14 (82.4)	2 (11.8)	1 (5.9)	0 (0.0)
Non-prescription illicit drug user	31	29 (93.5)	0 (0.0)	1 (3.2)	1 (3.2)
Gender					
Woman	66	53 (80.3)	10 (15.2)	1 (1.5)	2 (3.0)
Man	163	139 (85.3)	17 (10.4)	2 (1.2)	5 (3.1)
Transgender	2	2 (100.0)	0 (0.0)	0 (0.0)	0 (0.0)
Two-spirit	9	7 (77.8)	0 (0.0)	0 (0.0)	2 (22.2)
Queer	12	12 (100.0)	0 (0.0)	0 (0.0)	0 (0.0)
Non-binary	4	4 (100.0)	0 (0.0)	0 (0.0)	0 (0.0)
Agender	0	0 (0.0)	0 (0.0)	0 (0.0)	0 (0.0)
Other	1	1 (100.0)	0 (0.0)	0 (0.0)	0 (0.0)
MSM***	164	141 (86.0)	17 (10.4)	1 (0.6)	5 (3.0)
Ethnicity					
White	139	115 (82.7)	20 (14.4)	1 (0.7)	3 (2.2)
BIPOC	115	100 (87.0)	7 (6.1)	2 (1.7)	6 (5.2)
Ethnicity subgroup					
African, Caribbean or Black community	30	26 (86.7)	1 (3.3)	0 (0.0)	3 (10.0)
Person from the Indigenous community	12	11 (91.7)	1 (8.3)	0 (0.0)	0 (0.0)
Migrant	13	13 (100.0)	0 (0.0)	0 (0.0)	0 (0.0)
Received HIV diagnosis					
≤ 4 years ago	38	27 (71.1)	10 (26.3)	0 (0.0)	1 (2.6)
5–9 years ago	37	23 (62.2)	12 (32.4)	1 (2.7)	1 (2.7)
10–14 years ago	41	37 (90.2)	3 (7.3)	0 (0.0)	1 (2.4)
≥ 15 years ago	134	126 (94.0)	2 (1.5)	2 (1.5)	4 (3.0)
On AVR**** medication	253	215 (85.0)	27 (10.7)	3 (1.2)	8 (3.2)
Comorbidity					
Hepatitis C	14	8 (57.1)	5 (35.7)	1 (7.1)	0 (0.0)
Diabetes	38	29 (76.3)	7 (18.4)	1 (2.6)	1 (2.6)
Kidney failure	14	5 (35.7)	8 (57.1)	1 (7.1)	0 (0.0)
Chronic liver disease	15	9 (60.0)	6 (40.0)	0 (0.0)	0 (0.0)
Chronic lung disease	17	12 (70.6)	5 (29.4)	0 (0.0)	0 (0.0)
Severe asthma	20	17 (85.0)	3 (15.0)	0 (0.0)	0 (0.0)
Smoking					
Yes	57	46 (80.7)	8 (14.0)	2 (3.5)	1 (1.8)
Not currently/in the past	96	84 (87.5)	9 (9.4)	1 (1.0)	2 (2.1)
Never	97	86 (88.7)	9 (9.3)	0 (0.0)	2 (2.1)
Vaping					
Yes	32	24 (75.0)	7 (21.9)	0 (0.0)	1 (3.1)
Not currently/in the past	36	27 (75.0)	7 (19.4)	2 (5.6)	0 (0.0)
Never	182	165 (90.7)	12 (6.6)	1 (0.5)	4 (2.2)
Smoking cannabis					
Yes	65	57 (87.7)	5 (7.7)	0 (0.0)	3 (4.6)
Not currently/in the past	80	68 (85.0)	10 (12.5)	2 (2.5)	0 (0.0)
Never	101	86 (85.1)	12 (11.9)	1 (1.0)	2 (2.0)
Consumption of cannabis or cannabinoid-based products (form other than smoking)					
Yes	58	49 (84.5)	6 (10.3)	1 (1.7)	2 (3.4)
Not currently/in the past	62	53 (85.5)	8 (12.9)	1 (1.6)	0 (0.0)
Never	128	111 (86.7)	13 (10.2)	1 (0.8)	3 (2.3)

Legend: Data presented as n(%),* PREFER NOT TO ANSWER, **HS = high school, ***MSM = men having sex with men, percent represented as MSM/males, ****AVR = antiviral therapy

**Table 2:** Odds Ratios of receiving at least one dose of COVID-19 vaccine (unadjusted analysis)

Factor	Odds ratio	95% Confidence interval	p-value
Sex (Male vs. Female)	1.42	[0.60, 3.34]	0.42
Age /per 10-year	2.55	[ 1.71, 3.82]	< 0.0001
Person ≥ 65 years of age (Yes vs. No)	1.82	[ 0.32, 10.39]	0.50
Education - at least with completed university (Yes vs. No)	2.33	[1.02, 5.32]	0.04
Person who injects drugs (Yes vs. No)	0.85	[0.18, 3.98]	0.84
Person who uses non-prescription illicit drugs (Yes vs. No)	8.52	[0.48, 150.59]	0.14
Persons of African, Caribbean or Black communities (Yes vs. No)	2.42	[0.43, 13.57]	0.32
Person from the Indigenous community (Yes vs. No)	0.98	[0.16, 6.00]	0.98
MSM (Yes vs. No) * – Male participants only	0.56	[0.10, 3.29]	0.52

Legend of table: *MSM = men who have sex with men

### Vaccine Hesitancy Scale and Confidence in COVID-19 Vaccine

**Fig. 1:**
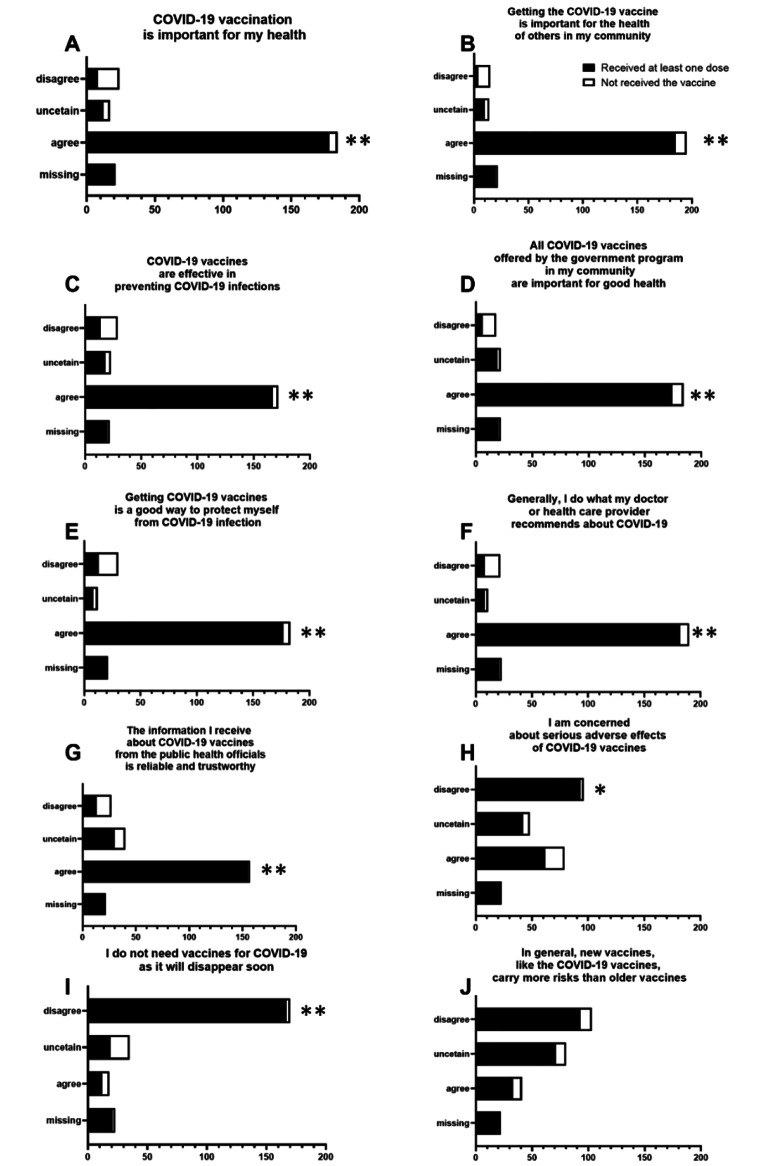
**Vaccine Hesitancy Scale (VHS) results based on COVID-19 vaccine uptake status. A-J** Overall patterns of vaccine hesitancy across the two groups based on the vaccine uptake presented by question. Five-point Likert scale ranging from 1 (strongly agree) to 5 (strongly disagree) are grouped into three categories, “agree”, “uncertain” and “disagree”. In the presence of a missing response, the total score was not calculated and set as missing. P values refer to the statistically different answers between vaccinated and not vaccinated individuals (**p < 0.0001, *p = 0.001)

The responses in VHS were grouped into three categories, “agree”, “uncertain” and “disagree” (Fig. 1). A total of 219 participants received at least one dose and 27 did not. The mean total VHS (SD) for persons having received at least one COVID-19 vaccine was 17.8 (6.2) compared to 35.4 (9.4) for participants not having received any COVID-19 vaccine (Supplemental Table 3 A). Participants accepted COVID-19 vaccines for both altruistic (i.e., protection of community) and individual reasons (i.e., protection of self). 89% of participants who received the vaccine considered COVID-19 vaccination to be important for their health, while 93% considered vaccination important for the health of others in the community. 87% trusted all COVID-19 vaccines offered by the government program and 92% trusted their health care provider recommendations. 90% believed that receiving COVID-19 vaccines offers good protection against COVID-19 infection, while 31% of participants who received the vaccine were concerned about serious adverse effects. Participants were divided on the risks that newly developed vaccines (e.g. COVID-19 vaccines) carry compared to older vaccines (18% agree, 36% uncertain and 46% disagree) regardless of their vaccination status. Individuals who felt that the pandemic would linger on longer were more likely to accept the vaccine (84.3% of vaccinated participants) than those who did not feel the pandemic would last that long (24% of unvaccinated).

Univariate logistic regression analyses revealed that the odds of taking at least one vaccine dose were increased 2.55 fold [95% CI 1.71, 3.82] with each increase in age of 10 years (p < 0.0001). No effect was observed for sex, belonging to the BIPOC or MSM community or use of injectable or non-prescription illicit drugs. We ran models including all significant variables from the univariate analysis (including and excluding VHS score). In both analyses, age, either as a continuous variable or dichomotomous variable (over 40 versus under 40), was identified as a risk factor. Multivariate logistic regression analyses revealed an odds of taking at least one vaccine dose were increased 2.4 fold [95% CI 1.6, 3.5] with each increase in age of 10 years (p<0.0001). The impact of age (over 40 versus under 40 years) was diminished but still significant when VHS was considered. Of note, we preferred the model without VHS because VHS is assumed to be on the causal pathway – older patients are less vaccine hesitant and hesitancy drives vaccination status.

## Discussion

There is limited data related to factors associated with COVID-19 vaccine confidence in PLWH living in Canada. Our study was based on the The Theory of Planned Behavior, which aims to provide a rationale for behaviors over which people can exert self-control[20, 21]. The key component to this model is behavioral intent, in that behavioral intentions are influenced by the attitude about the likelihood that the behavior will have the expected outcome and the weighing of the risks and benefits of that outcome. Our study was done later in the pandemic, after a lot of assurance from the literature on the safety of the vaccine and evidence of their effectiveness in preventing hospitalization and death. We found that older PLWH were more likely to accept COVID-19 vaccines. In addition, PLWH accept COVID-19 vaccines for both altruistic and individual reasons.

To our knowledge, the only other study conducted on vaccine uptake in PLWH living in Canada was that performed by Kaida et al., where the intention to receive COVID-19 vaccination was examined in HIV-positive and HIV-negative women and gender-diverse individuals in British Columbia (from August 2020 to March 2021)[42]. Their results indicated that HIV status itself was not associated with COVID-19 vaccine intention in adjusted analyses. In the HIV group, as in our study, older PLWH were more likely to report an intention to vaccinate [42]. Additionally, positive attitudes towards the COVID-19 vaccine were more strongly influenced by direct and indirect social norms toward vaccination[42] – something that we did not examine in our survey. Using an online survey, in an analysis restricted to women and gender-diverse individuals (n = 5588), they found only 65% of PLWH reported intention to receive a COVID-19 vaccine if recommended to them, compared to people who did not have HIV (80%)[40]. Of note, HIV itself was not associated with COVID-19 vaccine intention in adjusted analyses. Among PLWH, positive attitudes towards the COVID-19 vaccine were more strongly influenced by direct and indirect social norms toward vaccination. Similar to our study, PLWH with a higher odds of reporting intention to vaccinate were older[40].

Among other published studies in PLWH performed outside of Canada, Govere-Hwenje et al. also found that older age was associated with willingness to accept vaccination[35]. Telephone interviews were conducted with a randomly selected subset of participants enrolled in an observational cohort study evaluating decentralized ART delivery in South Africa (n = 213). Close to half of all individuals were unsure or were unwilling to be vaccinated[35]. Higher medical mistrust related to COVID-19 and the use of social media for COVID-19 information were associated with a lower willingness to accept vaccination[35]. Age and educational level were examined in an online survey conducted by Zheng et al. on 1295 MSM living with HIV (median age 29 years) residing in mainland China. Uptake of COVID-19 vaccine was only 9%[43]. In contrast to our study, age was not correlated with COVID-19 vaccine hesitancy[43]. However, in line with our study, education level was not associatetd with COVID-19 vaccine hesitancy [43]. Interestingly, Zheng et al. reported that concern about disclosure of HIV status was one of the two top reasons not to initiate COVID-19 vaccination in the study[43].

Factors facilitating willingness to be vaccinated amongst high-risk populations include perceptions of personal susceptibility[15]. Older persons likely perceive themselves to be at greater risk of worse outcomes from COVID-19 infection than younger individuals. In PLWH, greater hesitancy was observed among those who did not perceive their health status to have a major influence on their risk of illness associated with COVID-19[31]. In another survey conducted among 267 PLWH in France, Vallée et al. found that 30% of respondents endorsed hesitancy toward COVID-19 vaccination[36]. They also identified concerns about self-health, the requirement of mandatory COVID-19 vaccination, and personal chronic disease status as independently associated with vaccine acceptance[36]. Participants presenting with general vaccine refusal endorsed concerns about the vaccine and included those who believed they had already developed immunity to COVID-19[36].

Although we did not observe an effect of educational level on COVID-19 vaccine uptake, persons who have obtained higher levels of education may be more comfortable critically evaluating health information and deciding which sources of information are reliable. Similarly, the role of health literacy is known to affect vaccine uptake, although it has not been examined in the context of COVID-19 vaccine uptake in PLWH. Context plays an important role when considering vaccine confidence. With this in mind, not all studies have found that age or education is associated with vaccine hesitancy status. In a study involving 438 PLWH living in India who participated in telephone interviews, over one-third of participants were deemed “vaccine hesitant”, indicating that they were either unlikely to get vaccinated or that they wanted to wait[44]. While neither age nor education was associated with vaccine hesitancy in this study, vaccine hesitancy was associated with a lack of confidence in vaccine safety, concerns about side effects and efficacy, and lack of confidence in common sources of vaccine-related information such as doctors, news media, government[44].

In our study, PLWH were motivated to accept the COVID-19 vaccine for both personal and altruistic reasons. Altruism has previously been demonstrated as a reason for behavior in PLWH including the willingness to participate in research studies even when some personal risk is involved, such as HIV therapeutic vaccine trials[45]. Fostering altruism and responsibly promoting the societal benefits of vaccination may facilitate vaccine uptake amongst PLWH.

Most studies on vaccine confidence conducted in PLWH have been cross-sectional surveys. In contrast, Iliyasu et al. conducted a mixed methods cross-section study administering questionnaires to adults in Nigeria. This was complemented with 20 in-depth interviews of approximately half of respondents willing to receive the COVID-19 vaccine [46]. Some findings were similar to ours - acceptability of the COVID-19 vaccine was influenced by risk perception and concern about vaccine safety. Unlike our study, sociodemographic variables (sex, ethnicity) were not associated with COVID-19 vaccine acceptance. Themes revealed doubts about the existence of COVID-19, mistrust of authorities, and popular credence to rumors and conspiracy theories[46]. In conclusion, COVID-19 vaccine acceptance was sub-optimal and influenced by respondents’ age, income, co-morbidities, risk perception, and concerns about vaccine safety, efficacy, and rumours.

Possible reasons for discrepancy across studies include timing of study completion considering that vaccine confidence is an evolving phenomenon. In studies published earlier during the vaccine roll-out, those believing that the COVID-19 vaccine would not be effective in preventing COVID-19 infection may have reported greater hesitancy. However, as the pandemic progressed, evidence regarding the vaccine’s safety and efficacy emerged, which may have increased vaccine confidence in persons who completed surveys later during the pandemic. Furthermore, social norms may affect individuals’ responses when survey questions are answered anonymously via an online survey compared to when questions are asked by research staff. Enforcing social norms encourages behaviors perceived as “normal” or the proper thing to do[47, 48]. Although social norms play a role in vaccine uptake[49, 50], their influence in modifying vaccine hesitancy in PLWH towards COVID-19 vaccination has not been explored.

Disparity in some responses gathered in our evaluation compared to surveys from other countries may be explained by the impact of different levels of HIV-related stigma. Furthermore, mistrust of government likely differs based on the country and type of government in which the study was conducted. Stigma may have been a barrier to HIV management during the pandemic, by forcing individuals to disclose their HIV status to travel to the clinic during times of COVID-19-related travel restrictions. In a study of over 303 South African participants living with HIV, scales assessing medical mistrust, conspiracy beliefs, anticipated and internalized stigma and stereotypes specific to COVID-19 were applied. Greater than 50% of individuals agreed or strongly agreed, with at least one item suggestive of stigma, and more than 40% with an item assessing mistrust. Higher scores were associated with the female gender and a history of HIV-related stigma[28]. In another study conducted in Black Americans living with HIV, 101 individuals completed telephone interviews on negative impacts of COVID-19: general COVID-19 mistrust, COVID-19 vaccine and treatment hesitancy, and trust in COVID-19 information sources[30]. Greater COVID-19 mistrust was associated with greater vaccine and treatment hesitancy. Participants with more negative COVID-19 impacts reported lower ART adherence as well[30]. Therefore, stigma and medical mistrust may exacerbate pre-existing HIV stigma and medical mistrust on health protective behaviors in PLWH and especially ethnic minorities or persons of colour[30].

There are limitations to our study. PLWH are a very heterogeneous group, with different representation, volunteer and social desirability biases. We had difficulty recruiting participants in the prairie provinces and the Maritimes, in addition to some sub-populations that face social inequities, such as Indigenous persons. Therefore, the findings of the survey may not reflect all PLWH in Canada. Similarly, many more respondents lived in urban than in rural settings. Furthermore, adding focus groups whereby participants can exchange ideas and opinions amongst themselves may have highlighted other aspects of vaccine confidence which were not captured via the web-based e-questionnaire and provided richer data.

In the general public, educational interventions have been successful in increasing vaccine uptake for various infectious diseases[51, 52]. Understanding factors influencing COVID-19 vaccine uptake is critical to optimizing vaccine acceptance and preventing outbreaks. This is especially true for those experiencing social and health inequities. Persons with chronic disease, including PLWH, tend to have close relationships with their health care providers and this opportunity can be used for vaccination education. The provision of special services to offer dedicated and flexible vaccination, coupled with education about vaccination, has been shown to positively impact influenza vaccine uptake in PLWH. Provision of vaccination clinics and vaccine education by staff they trust at the clinics where PLWH are followed may help to increase vaccine uptake amongst PLWH and minimize stigma.

## Conclusion

PLWH accept COVID-19 vaccines for altruistic and individual reasons. Interventions to promote vaccine uptake amongst PLWH should be culturally and context-appropriate tailored. Older PLWH were more likely to receive at least one dose of a COVID-19 vaccine. Additional work is required to examine sustainability of behaviour change beyond public health and other mandates. With evolving recommendations and increasing numbers of booster vaccines, we must re-examine the needs of PLWH on an regular basis. We must also consider how to transfer these findings to promote uptake of influenza and other vaccinations.

## Electronic Supplementary Material

Below is the link to the electronic supplementary material.

Supplementary Material 1

Supplementary Material 2

Supplementary Material 3

Supplementary Material 4

## Data Availability

All relevant data are within the paper and its supporting files.

## Declarations

### Author Information

CTC is supported by a Fonds de recherche du Québec-Santé (FRQS) Junior 2 career award. ANB holds a Canada Research Chair (Tier 2) in Sexually Transmitted Infection Prevention. WZ is supported by the Michael Smith Health Research BC Scholar Program.

### Clinical Trial Number

N/A.

## List of Abbreviations

ART: antiretroviral therapy
BIPOC: Black, indigenous and people of colour
MSM: men who have sex with men
PLWH: persons living with HIV
VHS: Vaccine Hesitancy Scale
