# Supplementary material for: Understanding COVID-19 Vaccine Confidence in People Living with HIV: A pan-Canadian Survey
Source: AIDS Behav. 2023 Feb 4;27(8):2669–80. doi: 10.1007/s10461-023-03991-8 (PMC9898854; doi:10.1007/s10461-023-03991-8)
Supplement: Supplementary file 1 — Supplementary Material 1 [file 10461_2023_3991_MOESM1_ESM.docx]

**Supplemental Table 2: Demographics of participants based on COVID-19 vaccine uptake**

|  | total (n) | Vaccinated (n) | Unvaccinated (n) | % of total | % of vaccinated |
| --- | --- | --- | --- | --- | --- |
| Age ≥40 years old | 167 | 162 | 5 | **64.5** | 74.0 |
| Male | 180 | 162 | 18 | **69.5** | 74.0 |
| Gender (man) | 156 | 139 | 17 | **60.2** | 63.5 |
| Gender (woman) | 63 | 53 | 10 | **24.3** | 24.2 |
| Transgender, Two-spirit, Queer, Non-binary, agender | 25 | 25 | 0 | **9.7** | 11.4 |
| Less than high school | 5 | 4 | 1 | **1.9** | 1.8 |
| Some/ completed high school | 50 | 36 | 14 | **19.3** | 16.4 |
| Some/ completed university | 143 | 137 | 6 | **55.2** | 62.6 |
| Some/ completed graduate school | 47 | 41 | 6 | **18.1** | 18.7 |
| Employed | 136 | 120 | 16 | **52.5** | 54.8 |
| Born in Canada | 157 | 132 | 25 | **60.6** | 60.3 |
| MSM*** | 158 | 141 | 17 | **61.0** | 64.4 |
| White | 135 | 115 | 20 | **52.1** | 52.5 |
| BIPOC | 107 | 100 | 7 | **41.3** | 45.7 |
| Received HIV dg<15 | 112 | 87 | 25 | **43.2** | 39.7 |
| Received HIV dg≥15 | 128 | 126 | 2 | **49.4** | 57.5 |
| On AVR**** medication | 242 | 215 | 27 | **93.4** | 98.2 |
| Hepatitis C | 13 | 8 | 5 | **5.0** | 3.7 |
| Diabetes | 36 | 29 | 7 | **13.9** | 13.2 |
| Kidney failure | 13 | 5 | 8 | **5.0** | 2.3 |
| Chronic liver disease | 15 | 9 | 6 | **5.8** | 4.1 |
| Chronic lung disease | 17 | 12 | 5 | **6.6** | 5.5 |
| Inject drugs user | 16 | 14 | 2 | **6.2** | 6.4 |
| Current Smoker | 54 | 46 | 8 | **20.8** | 21.0 |
| Smoking cannabis | 62 | 57 | 5 | **23.9** | 26.0 |
