# Supplementary material for: Understanding COVID-19 Vaccine Confidence in People Living with HIV: A pan-Canadian Survey
Source: AIDS Behav. 2023 Feb 4;27(8):2669–80. doi: 10.1007/s10461-023-03991-8 (PMC9898854; doi:10.1007/s10461-023-03991-8)
Supplement: Supplementary file 2 — Supplementary Material 2 [file 10461_2023_3991_MOESM2_ESM.docx]

**Supplementary Table 3**

Supplementary Table 3A : Summary statistics for VHS total score by vaccination status

| **variable** | **Summary statistics** | COVID-19 vaccine uptake | |
| --- | --- | --- | --- |
|  |  | **Yes** | **No** |
| VHS_TOTAL | n | 194 | 18 |
|  | Missing, n (%) | 0 (0.0) | 0 (0.0) |
|  | Mean (SD) | 17.8 (6.2) | 35.4 (9.4) |
|  | Median (IQR) | 16.0 (14.0, 21.0) | 36.5 (31.0, 42.0) |
|  | Range | (10.0, 43.0) | (16.0, 48.0) |
|  |  |  |  |

| COVID-19 vaccine uptake | Estimate of mean difference | 95% confidence interval | p-value |
| --- | --- | --- | --- |
| Yes vs. No | -16.1 | [ -19.2, -13.0] | <0.0001 |

Supplementary Table 3B: Results of linear regression model (adjusted for age and sex)
