# Supplementary material for: Understanding COVID-19 Vaccine Confidence in People Living with HIV: A pan-Canadian Survey
Source: AIDS Behav. 2023 Feb 4;27(8):2669–80. doi: 10.1007/s10461-023-03991-8 (PMC9898854; doi:10.1007/s10461-023-03991-8)
Supplement: Supplementary file 3 — Supplementary Material 3 [file 10461_2023_3991_MOESM3_ESM.docx]

**Supplement Table 1**: Ten questions from the Vaccine Hesitancy scale (VHS) adapted to COVID-19

| **Ten questions/items on VHS** |
| --- |
| 1-COVID-19 vaccination is important for my health |
| 2- Getting the COVID-19 vaccine is important for the health of others in my community |
| 3-COVID-19 vaccines are effective in preventing COVID-19 infections |
| 4- All COVID-19 vaccines offered by the government program in my community are important for good health |
| 5- Getting COVID-19 vaccines is a good way to protect myself from COVID-19 infection |
| 6- Generally, I do what my doctor or health care provider recommends about COVID-19 vaccines for my health |
| 7- The information I receive about COVID-19 vaccines from the public health officials is reliable and trustworthy |
| 8- I am concerned about serious adverse effects of COVID-19 vaccines |
| 9- I do not need vaccines for COVID-19 as it will disappear soon |
| 10- In general, new vaccines, like the COVID-19 vaccines, carry more risks than older vaccines |
