# Supplementary material for: Understanding COVID-19 Vaccine Confidence in People Living with HIV: A pan-Canadian Survey
Source: AIDS Behav. 2023 Feb 4;27(8):2669–80. doi: 10.1007/s10461-023-03991-8 (PMC9898854; doi:10.1007/s10461-023-03991-8)
Supplement: Supplementary file 4 — Supplementary Material 4 [file 10461_2023_3991_MOESM4_ESM.docx]

**Supplemental Figure 1**: Participant flow chart
